# Spatially Segregated Transmission of Co-Occluded Baculoviruses Limits Virus–Virus Interactions Mediated by Cellular Coinfection during Primary Infection

**DOI:** 10.3390/v14081697

**Published:** 2022-07-31

**Authors:** Verónica Pazmiño-Ibarra, Salvador Herrero, Rafael Sanjuan

**Affiliations:** 1 Institute for Integrative Systems Biology (I2SysBio), Consejo Superior de Investigaciones Científicas-Universitat de València, C/Catedrático Agustín Escardino 9, 46980 Paterna, Spain; veronica.pazmino@uv.es; 2 Department of Genetics and Institute BIOTECMED, Universitat de València, 46100 Burjassot, Spain; salvador.herrero@uv.es

**Keywords:** baculovirus, occlusion bodies, collective infectious units, cooperation, social evolution

## Abstract

The occlusion bodies (OBs) of certain alphabaculoviruses are polyhedrin-rich structures that mediate the collective transmission of tens of viral particles to the same insect host. In addition, in multiple nucleopolyhedroviruses, occlusion-derived virions (ODVs) form nucleocapsid aggregates that are delivered to the same host cell. It has been suggested that, by favoring coinfection, this transmission mode promotes evolutionarily stable interactions between different baculovirus variants. To quantify the joint transmission of different variants, we obtained OBs from cells coinfected with two viral constructs, each encoding a different fluorescent reporter, and used them for inoculating *Spodoptera exigua* larvae. The microscopy analysis of midguts revealed that the two reporter genes were typically segregated into different infection foci, suggesting that ODVs show limited ability to promote the co-transmission of different virus variants to the same host cell. However, a polyhedrin-deficient mutant underwent inter-host transmission by exploiting the OBs of a fully functional virus and re-acquired the lost gene through recombination, demonstrating cellular coinfection. Our results suggest that viral spatial segregation during transmission and primary infection limits interactions between different baculovirus variants, but that these interactions still occur within the cells of infected insects later in infection.

## 1. Introduction

Collective infectious units are groups of viral particles that are jointly delivered to the same individual host and, in many cases, to the same cell within a host [1]. They have been found in widely different viruses, and include different types of structures, such as virion aggregates, extracellular vesicles containing pools of virions, and even cells carrying virions at their surface, such as gut microbiota [1,2,3,4,5]. A well-characterized type of collective infectious unit are the occlusion bodies (OBs) of lepidopteran nucleopolyhedroviruses (NPVs) (Family *Baculoviridae*, genus *Alphabaculovirus*), which comprise between one and tens of nucleocapsids embedded in a polyhedrin-rich proteinaceous matrix. OBs are produced in terminally infected larvae and are required for horizontal transmission through the oral route. OBs ingested by larvae are dissolved in the alkaline midgut, releasing occlusion-derived viruses (ODVs). In single nucleopolyhedroviruses, ODVs contain one or very few nucleocapsids per envelope, whereas in multiple nucleopolyhedroviruses, ODVs often harbor pools of nucleocapsids sharing an external membrane [6,7,8]. Therefore, OBs promote the joint transmission of groups of viral particles to the same individual host and, in principle, the ODVs of multiple nucleopolyhedroviruses enable the co-transmission of nucleocapsid pools to the same cell. Infected midgut cells produce budding virions (BVs), which disseminate within larvae as free virions [9], causing a systemic infection and death. During late infection, viral nucleocapsids located in the cell nucleus are enveloped to form new ODVs and OBs [6,7,8,10]. Hence, each OB contains viruses derived from the same cell.

OBs confer stability to baculovirus particles, which is an important fitness component for environmentally transmitted viruses. In contrast, the fitness advantage of producing ODVs with multiple nucleocapsids is less obvious. This trait has no simple genetic basis, since several genes determining the number and type of ODVs occluded within OBs have been described [11]. Furthermore, the trait depends on host physiology and its evolutionary origins remain poorly understood [9]. It has been suggested that primary infection foci initiated by ODVs containing multiple nucleocapsids are more likely to progress towards the systemic stage, either because the viral replication cycle is accelerated, helping the virus overcome certain host defenses, or because some of the nucleocapsids released by multiple ODVs traffic directly to tracheal cells or the hemolymph in the absence of replication [8,12,13].

It has been shown that OBs can mediate the co-transmission of different baculovirus genetic variants to the same larva. For instance, field isolates of Spodoptera frugiperda nucleopolyhedrovirus often contain OBs with a mixture of genotypes, including large deletion mutants [14]. This diversity was characterized by deep sequencing of Autographa californica multiple nucleopolyhedrovirus (AcMNPV) isolates, revealing that 25% of the genomes had large deletions in different regions [15]. This was subsequently confirmed by long-read sequencing, showing that genome structural variants constitute a significant source of diversity in these viruses [16]. Some of these variants are defective mutants that are unable to be transmitted orally, but can nevertheless persist in the population by using the OBs provided by polyhedrin-encoding viruses or other essential proteins present in the same host [17,18,19]. Similar to what has been shown for other viruses [20], defective baculoviruses can function as social cheaters that proliferate at the expense of functional variants (“helpers”), negatively impacting mean viral population fitness [21]. Alternatively, these mutants could participate in cooperative interactions that enhance pathogenicity and transmissibility. OBs containing a mixture of a complete virus and defective mutants lacking essential genes, for instance the per oral infectivity factor, were found to exhibit a lower lethal doses than single-genotype OBs [22,23,24]. Coinfection allowed defective genomes to use membranes containing this infectivity factor produced by complete genomes present in the same cell [25]. However, in most, cases, the mechanisms underlying viral cooperativity have not been completely studied.

Here, we used microscopic analysis to investigate the co-transmission of different genetic variants of AcMNPV through ODVs and OBs by measuring cellular coinfection during primary infection. For this, we inserted GFP or Cherry reporters in the viral genome, obtained OBs from cells coinfected with these two variants, and used them to inoculate larvae. We found that individual infection foci in the insect midgut typically displayed one or the other reporter, but rarely both, and that this segregation was maintained until late infection. On the other hand, confirming previous results, a polyhedrin-defective variant was able to exploit the OBs of a complete virus present in the same host to achieve transmission, showing that cellular coinfection occurs in a fraction of the host cells. Overall, these findings suggest that cooperative or antagonistic interactions between different baculovirus variants take place at the discrete stages of the viral infection cycle, such as during OB production and inter-host transmission, but are less likely at other stages, such as primary and early systemic infection, due to intra-host spatial population structure.

## 2. Materials and Methods

### 2.1. Cell Culture and Insects

*Spodoptera frugiperda* Sf21 cells (Invitrogen) were cultured in Gibco^TM^ Grace’s medium, supplemented (Fisher Scientific) with 10% heat-inactivated fetal bovine serum (FBS) at 26 °C. *Spodoptera exigua* larvae were reared on an artificial diet [26] at 25 °C with 70% relative humidity and a photoperiod of 16/8 h (light/dark).

### 2.2. Construction of Recombinant Viruses

A plasmid called *pFBD_polh-polh-p10-eGFP* was provided by Dr. María Gabriela López (Instituto de Agrobiotecnología y Biología Molecular, INTA-CONICET). Plasmid *pFBD_polh-pSel-X-p10-eGFP*, which lacks the polyhedrin gene, was obtained previously in our laboratory [27]. Plasmid *pFBD_polh-polh-p10-mCherry* was constructed by PCR-amplifying the *mCherry* gene from a commercial plasmid with primers mCherry_F and mCherry_R (Supplementary Table S1). The mCherry amplicon was digested with *SmaI* and *NheI* and cloned under the p10 promoter into a plasmid *pFBD_polh-polh-p10-eGFP,* which was previously digested with the same restriction enzymes in order to remove the eGFP gene (*pFBD_polh-polh-p10-X).* Infectious particles were obtained with the Bac-to-Bac baculovirus expression system, following the manufacturer’s instructions. In brief, plasmids were transformed into *E. coli* DH10Bac heat shock competent cells. Then, recombinant baculoviruses expressing polyhedrin and eGFP (*BAC_polh-polh-p10-eGFP*) or mCherry (*BAC_polh-polh-p10-mCherry*), as well as *BAC_polh-psel-X-p10-eGFP*, which does not express polyhedrin, were generated by homologous recombination and verified by PCR and Sanger sequencing. The isolated bacmids were purified following the manufacturer’s instructions and used for transfecting Sf21 cells using Cellfectin II Reagent (Invitrogen) and incubated at 27 °C. Recombinant baculoviruses were harvested after 72 hpi, centrifuged at 500× *g* for 5 min, and filtered through a 0.2 μm filter. To obtain high-titer stocks, infection supernatants were concentrated by loading the supernatants into a 25% sucrose solution in containing 5 mM NaCl and 10 mM EDTA, and centrifuging at 80,000 g for 90 min. The BV-containing pellet was resuspended in Grace medium and stored at 4 °C.

### 2.3. Viral Titration

Viral titers were measured by the foci assay [28]. For this, serial dilutions of the virus were used to inoculate confluent Sf21 cells in 12-well plates (100 µL inoculum) and, after 2 h at room temperature, the inoculum was removed by aspiration and cells were cultured with Grace’s medium supplemented with 10% FBS, 10 units/mL penicillin, 10 µg/mL streptomycin, and 3% Sea Plaque agar. Wells were incubated at 27 °C for 5–6 days until fluorescent foci were observed. Imaging was performed in an IncuCyte S3 Live-Cell Analysis System (Essen BioScience) using phase contrast, green or red channels with a 10× objective.

### 2.4. Flow Cytometry

Infected Sf21 cells were collected by centrifugation at 500× *g* for 5 min, washed with PBS 1X, resuspended at a density of 10^6^ cells/mL in Grace’s medium (1X) containing 2% FBS, and loaded into a Becton Dickinson LSR Fortessa flow cytometer. We analyzed at least 10^5^ events for each sample using 488 nm and 561 nm emission wavelengths for GFP and Cherry, respectively. Non-infected cells and cells infected only with one variant of virus were used as controls. Data analysis was performed using FlowJo software.

### 2.5. OB Preparation

Infected Sf21 cultures were incubated for 6 days, such that most cells showed polyhedra within nuclei. OBs were purified according to the protocol described by Caballero et al. [29] with certain modifications. Cells and media were collected and centrifuged at 1000× *g* for 5 min, the supernatant was discarded, and cells were lysed with 0.1% SDS. The lysate was loaded onto a 40% sucrose solution, centrifuged at 30,000× *g* for 30 min, and the pellet was washed with sterile distilled water three times. OBs were resuspended in sterile distilled water and counted in a Neubauer chamber (0.100 mm/0.0025 mm^2^).

### 2.6. Transmission Electron Microscopy

OBs were fixed at 37 °C for 1 h with a 1:1 *v:v* 4% paraformaldehyde—5% glutaraldehyde in phosphate buffer (PB) 0.1 M and encased in a 1% agar cone. The fixative was removed by successive PB 0.1 M washes, post-fixed and stained with a 2% osmiun solution, and dehydrated in a graded series of ethanol. Finally, samples were embedded in Durcupan epoxy resin (Sigma, Burlington, MA, USA), which was allowed to polymerize at 70 °C for 72 h. Ultrathin sections (0.08 μm) were obtained using an ultramicrotome (Leica EM UC-6) with a diamond-tipped knife (Ultra 45o; Diatome) and stained with Reynold’s lead citrate and 2% uranyl acetate. Samples were observed under a transmission electron microscope (Jeol JEM-1010). Acquired images from 35 individual OBs were visualized with ImageJ software. Cross-section images were used to determine the number of ODVs per OB. The number of nucleocapsids per ODV was obtained using only transverse ODV sections, since in longitudinal sections some nucleocapsids may fall under or above the imaged field.

### 2.7. Midgut Infection Analysis

*S. exigua* larvae were reared at 26 °C and fed with artificial diet. Groups of between 30 and 40 third-instar larvae were infected by the droplet feeding method with a solution containing 10% sucrose, 1% PBS, phenol red, and 10^6^ OBs/mL. The inoculum concentration was previously determined in an independent bioassay using 10^6^, 10^7^, and 10^8^ OBs/mL and the whole gut was analyzed from 12 to 72 hpi, every 12 h. Entire guts were extracted from animals at 72 hpi, washed twice with PBS, and immediately observed under a Leica MZ10F fluorescence stereo microscope. A random selection of foci in each midgut was analyzed. Acquired images were analyzed with ImageJ, and the number of red, green, and doubly fluorescent infection foci was determined by manual counting. As indicated, some samples were checked by confocal microscopy in an Olympus FV1000 confocal laser scanning microscope. Larvae that died from virus infection were homogenized and frozen at −20 °C. The OBs obtained from these larvae were filtered through cheesecloth, washed twice with 0.1% SDS, and purified as described above. The resulting OBs were subsequently used for infecting a second generation (F2) of *S. exigua* larvae, and the midguts were analyzed as above.

### 2.8. Hemolymph-Derived BVs Extraction

The surface of larvae was cleaned with absolute ethanol and the hemolymph was collected by proleg incision with sterile surgical scissors. Approximately 10 µL of hemolymph was placed in a microtube containing ice-cold PBS (1X) to prevent melanisation. The diluted hemolymph was filtered using a 0.45 μm filter and stored at −20 °C. Viral titers were determined by the foci assay as above.

### 2.9. Determination of Coinfected Cells in Whole Larvae

Larvae were encased in cryomolds and coated with embedding medium for freezing (Tissue-Plus O.C.T). Immediately after, the molds were immersed in liquid nitrogen for a few seconds and then kept at −80 °C. Longitudinal 25 μm sections of 12 entire larvae were prepared using a cryostat (Leica CM1950). Slides with tissue sections were stored at −20 °C until use. Tissue samples were stained with DAPI (4′,6-diamidino-2-phenylindole), incubated for 10 min, and washed twice with PBS. The slides were mounted with FluorSave reagent under a coverslip and observed with a fluorescence microscope equipped with DAPI, GFP, and Cherry filters.

Acquired images were analyzed with ImageJ software, and the number of red, green, and doubly fluorescent cells was determined by manual counting.

### 2.10. PCR Detection of Spontaneous Recombinants

The hemolymph of F2 infected larvae (Section 2.7) was collected in 10 µL TE buffer (1X), treated with PrepMan Ultra reagent (Applied Biosystems), following the manufacturer instructions, and used for PCR amplification of the targeted region with specific primers (Supplementary Table S1). A first PCR was performed to validate the integrity of the extracted DNA using universal_p10-F and eGFP-F primers (Supplementary Table S1) that amplified a 404 bp fragment of the eGFP gene cloned under the p10 promoter. In a second PCR, eGFP-F and polyhedrin-R primers (Supplementary Table S1) were used for detecting recombinants containing both eGFP and polyhedrin (Supplementary Figure S3c). The expected size of this fragment was approximately 800 bp. In both cases, we used Phusion High-Fidelity DNA Polymerase (Thermo Scientific, Waltham, MA, USA) for PCR amplification under the following thermal profile: initial denaturation at 98 °C for 30 s, 35 amplification cycles of 98 °C for 10 s, annealing for 30 s at 60.9 °C for the universal_p10-F and eGFP-F primers pair or 66.8 °C for the eGFP-F and polyhedrin-R primer pair, and 72 °C for 40 s, and a final extension at 72 °C for 5 min. PCR products were visualized by agarose gel electrophoresis.

### 2.11. Foci Assay-Based Detection of Spontaneous Recombinants

The hemolymph of 14 infected larvae from the F2 generation was collected in 10 µL of ice-cold PBS (1X), filtered through a 0.45 μm membrane filter, and used directly for foci assays in Sf21 cells. Well-isolated foci were analyzed at 96 hpi under a fluorescent microscope equipped with GFP and Cherry filters and incubated an additional 48 h to check the production of polyhedrin in the phase contrast channel.

### 2.12. Calculation of Expected Coinfection Rates under Free Assortment

To calculate the expected fraction of mixed ODVs produced by Sf21 cells coinfected with GFP and Cherry variants (*P*), we used the distribution of the number of nucleocapsids per ODV (*f_n_*, defined as the fraction of ODVs containing *n* nucleocapsids), and the abundance of the GFP (*p*) and Cherry (*q* = 1 − *p*) variants relative to total viruses as estimated by BV titration, which yielded *p* = 0.547. Under the assumption of free assortment, $P=\overset{\infty }{\underset{n=1}{\sum }}f_{n}\left(1-p^{n}-q^{n}\right)$. We also calculated the expected fraction of mixed OBs produced by coinfected cells as $Q=\overset{\infty }{\underset{n=1}{\sum }}g_{n}\left(1-{\left(1-P\right)}^{n}\right)$, where *g_n_* is the fraction of OBs containing *n* ODVs. Assuming that midgut infection foci were initiated by one ODV each, the expected fraction of doubly fluorescent foci was *cP*, where *c* was the observed percentage of Sf21 producer cells coinfected with both variants (*c* ≈ 0.9).

## 3. Results

### 3.1. Production of OBs from Cells Coinfected with GFP- and Cherry-Encoding Viruses

**Figure 1:**
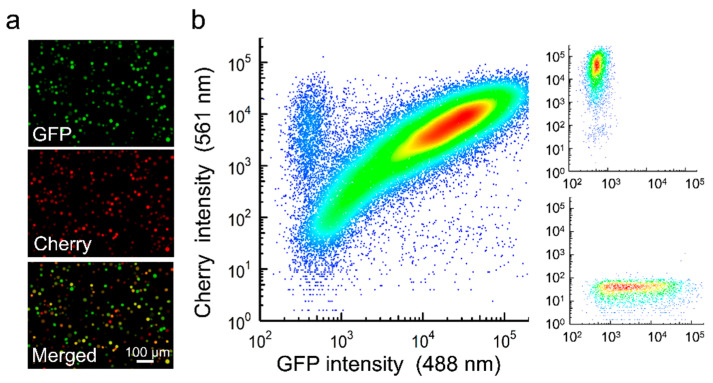
Coinfection of Sf21 cells with GFP- and Cherry-expressing AcMNPV. Cells were inoculated at an MOI of 5 PFU/cell and analyzed at 40 hpi. (**a**). Fluorescence microscopy. (**b**). Flow cytometry. Small panels on the right show monoinfected controls.

**Figure 2:**
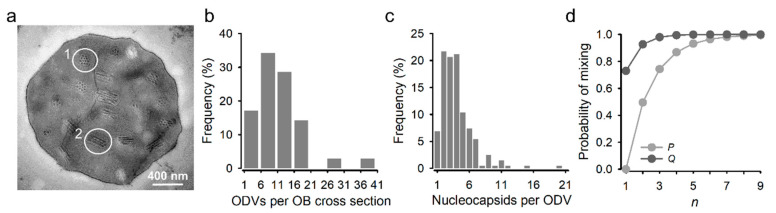
ODV and nucleocapsid content of AcMNPV OBs. (**a**). Transmission electron micrography of an OB obtained from Sf21 cells coinfected with the GFP- and Cherry-expressing variants at an MOI of 5 PFU/cell and harvested at 6 dpi. ODVs appear in transverse (1) and longitudinal (2) sections. (**b**). Distribution of the observed number of ODVs per OB cross section. (**c**). Distribution of the number of nucleocapsids per ODV. Only transverse sections were used for determining this distribution. (**d**). Probability that an ODV (*P*, light grey) or OB (*Q*, dark grey) contains a mixture of nucleocapsids from both types (GFP and Cherry) assuming free assortment of the two variants in coinfected cells. *P* depends on the number of nucleocapsids per ODV (shown as *n* in the x-axis). To calculate *Q*, we integrated *P* over the observed distribution of the number of nucleocapsids per ODV, shown in c. *Q* further depends on the number of ODVs per OB (also represented as *n* in the x-axis). See methods for calculation details.

Recombinant AcMNPVs carrying the Cherry or GFP gene were constructed using the Bac-to-Bac system, and BVs were recovered by transfection of Sf21 cells. After preparation of purified BVs of each type, Sf21 cells were simultaneously inoculated with both viral variants at an overall multiplicity of infection (MOI) of approximately five plaque forming units (PFU) per cell to promote cellular coinfection. Analysis of these cultures at 40 h post inoculation (hpi) by fluorescence microscopy revealed that 92.9 ± 0.1% of the cells expressed both reporters (Figure 1a). Similar results were obtained by flow cytometry (89.4 ± 1.2% coinfection; Figure 1b). BVs harvested from these cultures were titrated by the foci assay, yielding similar titers for GFP and Cherry viruses (8.1 × 10^5^ and 6.7 × 10^5^ PFU/mL, respectively). OBs were extracted at 6 days post inoculation (dpi), and 35 individual OBs were examined by transmission electron microscopy to characterize the distribution of the number of ODVs per OB cross section, and of the number of nucleocapsids per ODV (Figure 2a). On average, each OB cross section contained 11 ODVs, with 94.3% containing between 2 and 18 (Figure 2b). In turn, the examination of 203 ODVs within these OBs indicated that each ODV contained four nucleocapsids on average, with 89.7% of ODVs containing between two and nine (Figure 2c), consistent with the observed distribution for nucleocapsids per ODV in a previous study [30]. Based on these distributions, the observation that roughly 90% of producer cells were infected with both variants, and the relative frequency of GFP and Cherry viruses estimated by BV titration, we inferred that 65.7% of ODVs and 89.5% of OBs released by producer cells should harbor both reporters under the assumption of random assortment (Figure 2d).

### 3.2. Spatial Segregation of GFP and Cherry Variants In Vivo

**Figure 3:**
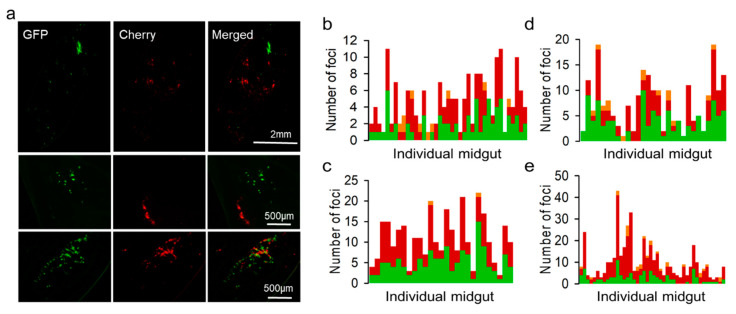
Microscopic examination of midgut infection foci at 72 hpi. (**a**). Representative images of full midguts (top) and individual foci showing segregated (middle) or co-localizing (bottom) GFP and Cherry reporters. (**b**). Analysis of larvae inoculated with OBs prepared from coinfected Sf21 cells (F1). Each bar represents the midgut of an individual larva and the stacked colors indicate the numbers of GFP-positive (green), Cherry-positive (red), and doubly fluorescent (orange) foci. (**c**). Analysis of larvae inoculated with F2 viruses (i.e., OBs released by F1). (**d**). Larvae inoculated with OBs prepared from Sf21 cells coinfected with a polyhedrin-defective GFP virus and a complete Cherry virus (F1). (**e**). Analysis of larvae inoculated with F2 viruses derived from the F1 polyhedrin-defective GFP virus and complete Cherry virus infection.

We used the OBs extracted from the above cultures to inoculate Spodoptera exigua larvae, and midguts were dissected and examined by stereo microscopy at different time points to quantify red, green, and doubly fluorescent infection foci. At 48 hpi, we found infection foci typically constituted by one or few fluorescent cells that were often difficult to identify (Supplementary Figure S1). At 72 hpi, foci were more clearly observed (Figure 3a), and the examination of 187 individual foci from 39 larvae showed that 38.5% were positive only for GFP, 56.2% were positive only for Cherry, and 5.3% were positive for both reporters (Figure 3b). Since images showing double fluorescence might potentially result from the superposition of foci located at different positions in the z-axis, we also analyzed 91 infection foci in the midguts of nine larvae by confocal microscopy (Supplementary Figure S2a). Confirming our above results, the two reporters co-localized in only 3.3% of the foci (Supplementary Figure S2b). These data contrast sharply with our above expectation that 65.7% of ODVs should carry both variants under the assumption of random assortment.

We then set out to test whether the GFP and Cherry viruses were co-transmitted to the following generation. For this, we harvested OBs from 15 cadavers, pooled them, and used them for inoculating another generation of larvae (F2). The midguts of 27 animals were analyzed at 72 hpi by fluorescence stereo microscopy, as above. We found green and red foci in all midguts. However, the examination of 412 infection foci from these larvae showed that 45.1% were positive only for GFP, 54.4% were positive only for Cherry, and only 0.48% were positive for both (Figure 3c). Hence, both virus variants were transmitted through OBs and reached the same hosts (in the same or different OBs) but were very rarely transmitted to the same midgut cells. Hence, opportunities for virus–virus interactions following transmission were limited by within-host spatial segregation.

**Figure 4:**
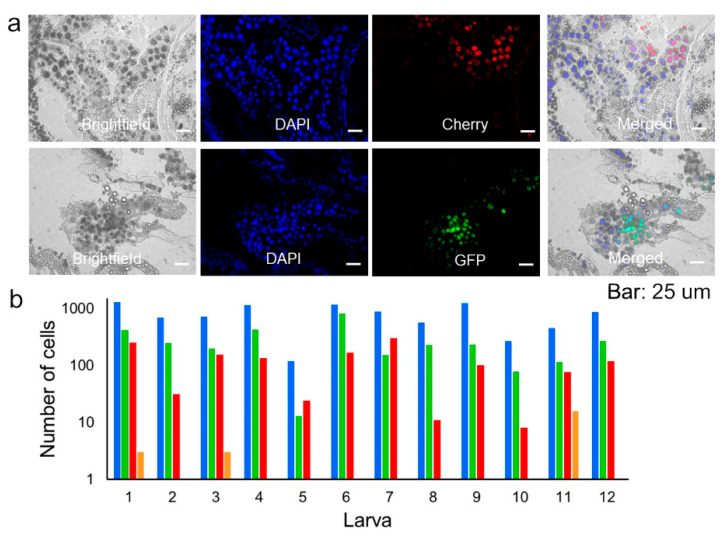
Analysis of variant mixing during late-stage infection. (**a**). Representative image of longitudinal sections of entire larvae inoculated with OBs from Sf21 cells coinfected with the GFP and Cherry variants (F1) obtained at 120 hpi. The phase contrast, DAPI, GFP, Cherry, and merged images are shown. Bars: 25 µm. (**b**). Counts for DAPI-positive (blue), DAPI + GFP-positive (green), DAPI + Cherry-positive (red), and triply positive cells (orange) for five larvae.

To better understand these results, we re-examined larva of the first generation (F1) at the latest stages of infection by performing longitudinal sections of 12 entire animals at 96 or 120 hpi (Figure 4a). Sections were stained with DAPI to count total cells. Out of 9390 DAPI-positive cells, 45.6 ± 4.2% expressed GFP or Cherry, indicating widespread infection. However, strikingly, only 0.76 ± 0.64% of infected cells were positive for both reporters, indicating that coinfection with the two variants was rare (Figure 4b). Therefore, the initial segregation observed in midguts was maintained throughout the infection process. As such, the low frequency of mixed foci in F2 (0.48%) was roughly consistent with the fraction of coinfected cells observed during late infection in F1 (0.76%).

### 3.3. Ability of a Polyhedrin-Defective Virus to Exploit OBs from a Functional Virus

We then set out to confirm previous findings, according to which transmission-defective viruses can exploit OBs produced by fully functional viruses present in the same host. To achieve this, we constructed a GFP-encoding virus defective for the polyhedrin gene, recovered and purified BVs, and used them for inoculating Sf21 cells at an MOI of approximately 5 PFU/cell with a mixture of this virus and the complete Cherry virus. The analysis of cultures at 40 hpi showed 72.6 ± 2.9% coinfection by fluorescence microscopy. OBs were extracted and used for inoculating larvae and counting midgut infection foci, as above. We counted 217 foci from 29 different larvae at 72 hpi, of which 50.7% were positive for GFP only, 45.2% for Cherry only, and 6.5% contained both reporters (Figure 3d). These percentages were similar to those obtained with fully functional viruses and further indicate that the polyhedrin-defective variant was efficiently loaded into OBs in the producer Sf21 cells. At 96 hpi, we extracted BVs from the hemolymph of six larvae and titrated these extracts by the foci assay, which yielded (1.4 ± 0.8) × 10^8^ and (1.1 ± 0.6) × 10^8^ PFU/mL for the polyhedrin-deleted GFP and the Cherry virus, respectively. Hence, both variants disseminated systemically with similar efficacy. We then obtained OBs from 15 cadavers, pooled them, and used them for inoculating new larvae. In this F2 generation, we examined 455 infection foci from the midguts of 44 different larvae. We observed GFP-positive cells in 41/44 larvae (93.2%) and Cherry-positive cells in all larvae. Overall, 23.5% of the foci were GFP-positive, 70.6% were Cherry-positive, and 5.9% contained both reporters (Figure 3e). This suggested that the polyhedrin-deficient virus was capable of undergoing larva-to-larva transmission, although it was less efficiently loaded into OBs than its polyhedrin-competent counterpart.

**Figure 5:**
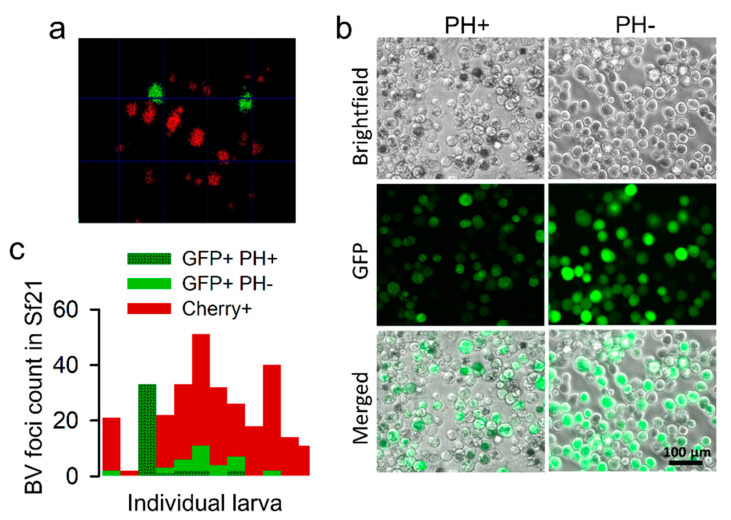
Detection of spontaneous recombinants by the foci assay. Serial dilutions of F2 BVs extracted from hemolymphs were used for inoculating Sf21 cultures, and individual foci were examined. (**a**). Representative image of GFP and Cherry foci at 96 hpi. (**b**). GFP-positive foci were further examined at 144 hpi by phase contrast and fluorescence microscopy to test for the absence (PH-) or presence (PH+) of OBs. (**c**). Foci count.

However, GFP-positive foci might also correspond to viruses that regained the polyhedrin gene through recombination with the complete virus. To test this, we obtained BVs from the hemolymph of 14 larvae infected with F2 viruses, extracted DNA, and used a pair of primers annealing with the GFP and polyhedrin genes to detect recombinants. We obtained amplicons of the expected size range in eight larvae (57.1%; Supplementary Figure S3a,b), suggesting that a fraction of the GFP-positive viruses in F2 were indeed recombinants. To investigate this more quantitatively, we used hemolymph-derived BVs to perform foci assays in Sf21 cells (Figure 5a), examined well-isolated foci by fluorescence microscopy to discern GFP- and Cherry-encoding viruses (Figure 5b), and incubated these cultures further to allow for the production of OBs. Out of 307 Sf21 infection foci isolated from 12 different larvae, 22.1% were GFP-positive and 77.9% were Cherry-positive (no doubly fluorescent foci were observed, as expected; Figure 5c). These percentages are consistent with those obtained by the examination of midgut infection foci in vivo (see above). Among the 68 GFP-positive foci, 58.8% showed OB production at 5 dpi or later, showing that these foci corresponded to recombinant viruses encoding GFP and polyhedrin. These results confirm that a polyhedrin-defective virus can exploit OBs from a complete virus for inter-host transmission but reveal that this process occurs with similar frequency as the recovery of the missing gene by recombination.

## 4. Discussion

Our strategy for investigating the co-transmission of genetically diverse baculoviruses through OBs and ODVs was based on the analysis of infection foci produced following viral entry through the oral route. The midgut analysis of F1 infected larvae showed that the fraction of mixed infection foci expressing both GFP and Cherry reporters was an order of magnitude lower than expected given random mixing (5.3% versus 65.7%), a result that did not vary appreciably depending on whether larva were inoculated with OBs carrying complete or polyhedrin-defective genomes in the first generation larvae analyzed (F1). This coinfection rate was low compared to the results obtained previously using midgut cells from another species [31].

Several factors could explain why we observed low levels of marker co-expression. First, ODVs with a single nucleocapsid would obviously lead to the formation of foci containing a single variant each. A previous publication described high frequencies of ODVs with a single nucleocapsid by electronic microscopy and physical separation [32], but this is in contrast to our results. Second, it is possible that the majority of nucleocapsids in an ODV are structurally defective and fail to undergo genome decapsidation and gene expression. Third, some ODV-derived nucleocapsids may traffic directly to tracheal cells or the hemolymph by budding through basal plasma membrane of midgut cells without replicating, as suggested previously [8,12,13]. Fourth, since the diameter of peritrophic membrane pores in midgut cells is similar to that of nucleocapsids [7], the size, and thus potentially, the number of nucleocapsids within ODVs could influence infection probability, disfavoring larger nucleocapsids that would tend to contain more genetic variants of the virus. Fifth, the ability of midgut cells to renew by sloughing and remove infected cells as a resistance mechanism [33,34], or delayed expression of genes related to OB production (*p10* and *polyhedrin*) [35] might have limited our ability to detect infection foci. It has been shown that genes controlled by late promoters, such as our reporters, tend to be expressed in a low fraction of infected cells [36]. This should reduce opportunities for co-expression of genes from different nucleocapsids, particularly if cells receiving multiple nucleocapsids tend to die faster than those receiving a single nucleocapsid.

In sum, several factors could limit the ability of viral genomes present in the same ODV to enter the same cell and share gene products. Regardless of the mechanisms at play, this would lead to variant segregation during primary infection and would prevent coinfection-dependent virus–virus interactions, such as genetic complementation, also removing a large fraction of defective viruses at this stage. In line with our findings, defective viruses that have been produced by high-MOI passaging in cell cultures are rapidly counter-selected in vivo [37], probably as a result of spatial segregation during primary infection.

Our experiments with complete (polyhedrin positive) viruses did not provide information about whether the two variants were packaged in the same OBs in the producer cells (co-occlusion), since OBs are dissolved in the midgut lumen and hence should not necessarily deliver their content to the same midgut cells. However, the assays performed with a polyhedrin-defective GFP-expressing virus and a functional Cherry virus confirmed that both genotypes infected the same cells, since the polyhedrin-defective virus alone would be unable to undergo oral transmission. Previous work has amply shown that OBs can be shared by different virus variants [14,17,18,19,21,22,23,24,38]. Mixing occurred both in Sf21 cells and in vivo, since the polyhedrin-defective virus was also transmitted to the F2 generation, albeit at lower frequency than in F1, suggesting more restricted OB sharing in vivo. Still, the transmission of this polyhedrin-defective variant was lower than in previous studies with *Trichoplusia ni* larvae [19,39], potentially due to differences in susceptibility between hosts. The persistence of the defective virus is influenced by the composition of the OBs, the proportion of each variant in the population, and the infection dose, since transmission bottlenecks could favor extinction of the defective virus [39]. The percentage of foci containing both GFP and Cherry markers in F2 infected larvae differed between assays performed with the complete virus (0.48%) and the defective (5.9%) virus. This difference could be due to the fact that coinfection is necessary to allow the defective variant to persist in successive rounds of infection, while coinfection is not essential for the maintenance of complete viruses.

The segregation of the GFP and Cherry variants into different midgut infection foci was observed in all experiments and suggests that the two variants were typically not packaged into the same ODVs, neither in cultured Sf21 cells nor in vivo. Previous reports have shown some extent of ODV co-packaging between virus variants present in cell cultures [18], between different alphabaculovirus species [40], and between virus variants present in single nucleocapsids and a multiple ODV [41], but our data suggest that this process occurs at low frequency. Based on this, we speculate that virus variants coinfecting the same cell often remain compartmentalized at the intracellular level. Baculovirus replication and encapsidation takes place inside nuclei in discrete structures called the virogenic stroma [8,42]. Imported viral DNA molecules might initiate a different replication and encapsidation center each, limiting admixture of different genetic variants in the same ODVs. Alternatively, it is possible that co-packaging in the same ODV occurred but that primary infection of midgut cells was typically initiated by only one nucleocapsid from each ODV.

Our analysis of late-stage infected larvae showed that the GFP and Cherry variants mixed infrequently despite widespread disseminated infection. This may explain why F2 midgut foci contained even fewer coinfected cells than F1 foci, but contrasts with previous work suggesting that the coinfection of individual cells by BVs of different virus variants is an important mechanism for the maintenance of genetic diversity in baculoviruses [19,43]. As a note of caution on our results, terminally infected larvae contained large numbers of dead cells. We indeed noticed high levels of green autofluorescence in these preparations, and this might have biased GFP-positive cell counts. Nevertheless, the numbers of doubly fluorescent cells were markedly lower than those of singly fluorescent cells, suggesting that coinfection was truly rare. This finding was apparently puzzling, considering that the MOI should be relatively high at this infection stage. We examined tissue samples from entire larva, including midgut tissue in which infection is typically not generalized, potentially leading to lower coinfection rates. Alternatively, our observations may be accounted for by superinfection exclusion, a mechanism displayed by most types of viruses whereby a resident virus impedes secondary infections of the same cell. In baculoviruses, superinfection exclusion is established as soon as 3 hpi becomes common after about 16 hpi or more and constitutes an efficient mechanism for preventing mixed infections [44].

Cellular coinfection necessarily occurs in vivo, since it is a condition for recombination and for the maintenance of defective viral genomes, both of which are well-known processes in baculoviruses and were observed in our experiments. In light of our results, we speculate that mixed infections are allowed, but only episodically and, maybe, specifically in certain cell types or at certain infection stages, such as for instance in some OB-producing cells. The relaxation of superinfection exclusion mechanisms might help promote these changes. In contrast, mixed infection avoidance might be less efficient in cell cultures, resulting in the faster production of recombinants and defective genomes compared to natural infections [45].

Overall, our findings suggest that the natural baculovirus infection cycle imposes certain barriers to interactions between different genetic variants, including genetic complementation and defective interference. In the social evolution field, it has been amply established that systematic mixing of genetically unrelated individuals makes cooperation unlikely, since under this scenario, selection favors cheater genotypes (including defective interfering viruses), which benefit from cooperators without reciprocating [46,47,48]. Spatial structure, such as the foci segregation we found in the midgut, increases genetic relatedness among potentially interacting viruses, making cooperation evolutionarily more stable. The different forms of spatial segregation have been reported in widely different viruses undergoing collective transmission, such as in enteroviruses transmitted through vesicles [49], HIV-1 spreading through viral synapses [50], and tomato mosaic virus spreading through plasmodesmata [51]. This suggests that collective infectious units do not favor cooperation between different genetic variants of a virus (e.g., genetic complementation). In contrast, the fitness advantage of spreading collectively could reside in other processes, thus increasing environmental stability, accelerating early infection stages, or evading cellular innate immune responses [2,52,53].

## Data Availability

The authors confirm that the data supporting the findings of this study are available within the article. The datasets used and/or analyzed during the current study are available from the corresponding author on reasonable request.

## Supplementary Materials

The following supporting information can be downloaded at: https://www.mdpi.com/article/10.3390/v14081697/s1, Figure S1: Microscopic examination of midgut infection foci at 48 hpi; Figure S2: Examination of midgut infection foci at 72 hpi by confocal microscopy; Figure S3: PCR-based identification of spontaneous recombinants; Table S1: PCR primers.
