# Supplementary material for: Spatially Segregated Transmission of Co-Occluded Baculoviruses Limits Virus–Virus Interactions Mediated by Cellular Coinfection during Primary Infection
Source: Viruses. 2022 Jul 31;14(8):1697. doi: 10.3390/v14081697 (PMC9413315; doi:10.3390/v14081697)
Supplement: Supplementary file 1 [file viruses-14-01697-s001.zip › viruses-1758090-Table S1.pdf]

viruses

Table S1. PCR primers

Primer	Sequence
**mCherry_F**	5'-GGTCTCGCGAGATGGTGAGCAAGGGCGAGGAG-3'
**mCherry_R**	5'-GGGCCGGCGAGACCCTACTTGTACAGCTCGTC-3'
**eGFP-F**	5'-TTGAAGAAGTCGTGCTGCTTCAT-3'
**Polyhedrin-R**	5'-ATCCTCAGCCACTAGGTAGTTGT-3'
**Universal_p10-F**	5'-CCTTTAATTCAACCCAACACAATA-3'
