# Supplementary figures and images for: Spatially Segregated Transmission of Co-Occluded Baculoviruses Limits Virus–Virus Interactions Mediated by Cellular Coinfection during Primary Infection
Source: Viruses. 2022 Jul 31;14(8):1697. doi: 10.3390/v14081697 (PMC9413315; doi:10.3390/v14081697)

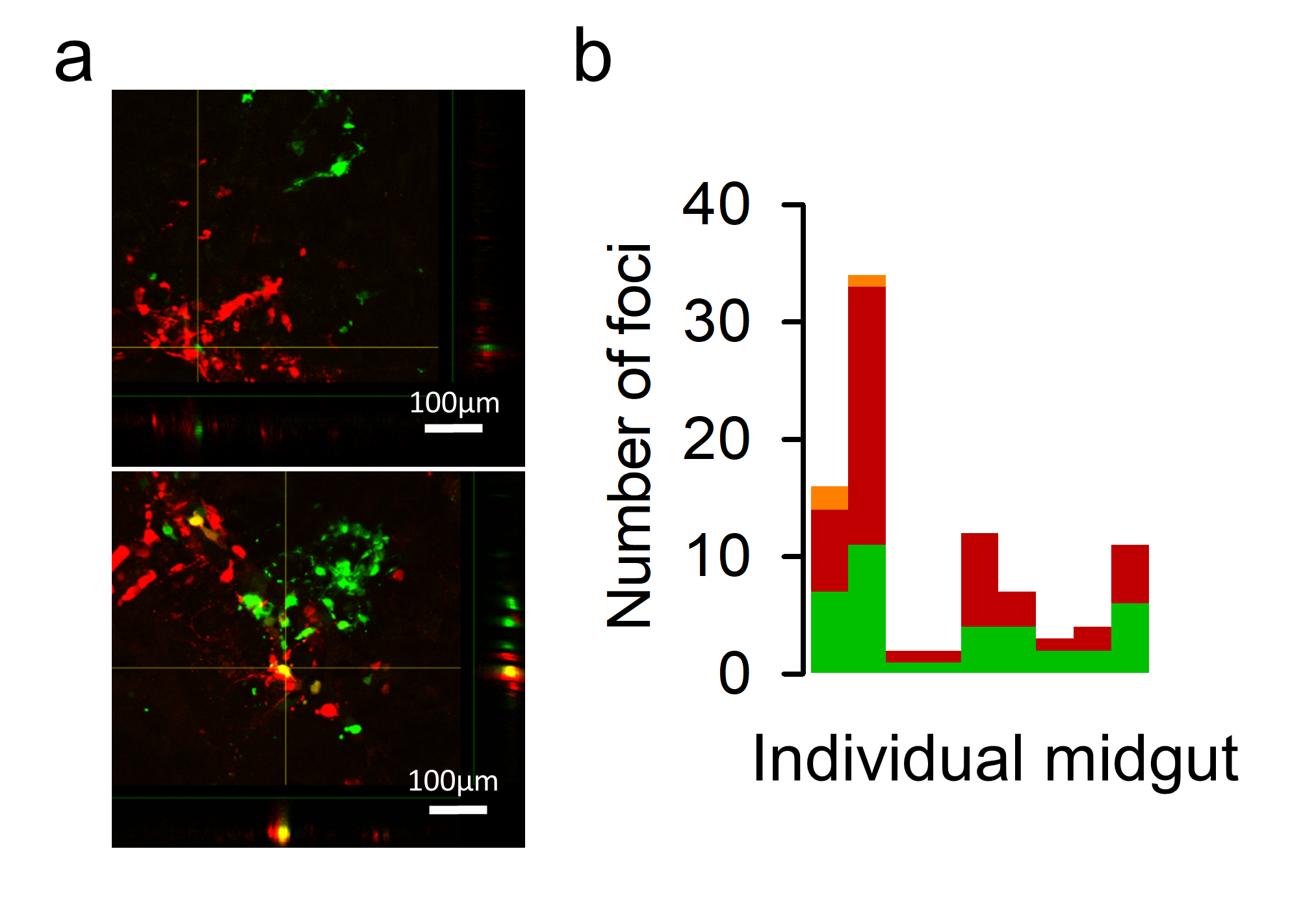

Supplement: Supplementary file 1 [file viruses-14-01697-s001.zip › FigureS2.tif]
